# Molecular characterization of irinotecan (SN-38) resistant human breast cancer cell lines

**DOI:** 10.1186/s12885-016-2071-1

**Published:** 2016-01-22

**Authors:** Haatisha Jandu, Kristina Aluzaite, Louise Fogh, Sebastian Wingaard Thrane, Julie B. Noer, Joanna Proszek, Khoa Nguyen Do, Stine Ninel Hansen, Britt Damsgaard, Signe Lykke Nielsen, Magnus Stougaard, Birgitta R. Knudsen, José Moreira, Petra Hamerlik, Madhavsai Gajjar, Marcel Smid, John Martens, John Foekens, Yves Pommier, Nils Brünner, Anne-Sofie Schrohl, Jan Stenvang

**Affiliations:** Faculty of Health and Medical Sciences, Department of Veterinary Disease Biology, Section for Molecular Disease Biology and Sino-Danish Breast Cancer Research Centre, University of Copenhagen, Strandboulevarden 49, DK-2100 Copenhagen, Denmark; Department of Pathology, Aarhus University Hospital, Noerrebrogade 44, building 18B, 8000 Aarhus C, Denmark; DTU Multiassay Core (DMAC), Technical University of Denmark, Kemitorvet Building 208, DK-2800 Lyngby, Denmark; Department of Molecular Biology and Genetics, Aarhus University, C.F. Møllers Allé 3, 8000 Aarhus C, Denmark; Brain Tumor Biology, Danish Cancer Society Research Center, Strandboulevarden 49, DK-2100 Copenhagen, Denmark; Erasmus MC Cancer Institute, Department of Medical Oncology and Cancer Genomics Netherlands, Erasmus MC, Rotterdam, The Netherlands; National Institutes of Health, National Cancer Institute, Center for Cancer Research, Developmental Therapeutics Branch and Laboratory of Molecular, Pharmacology, 37 Convent Drive, Building 37, Room 5068, Bethesda, MD 20892-4255 USA

**Keywords:** Breast cancer, Topoisomerase I, Irinotecan, SN-38, Resistance, ABCG2/BCRP

## Abstract

**Background:**

Studies in taxane and/or anthracycline refractory metastatic breast cancer (mBC) patients have shown approximately 30 % response rates to irinotecan. Hence, a significant number of patients will experience irinotecan-induced side effects without obtaining any benefit. The aim of this study was to lay the groundwork for development of predictive biomarkers for irinotecan treatment in BC.

**Methods:**

We established BC cell lines with acquired or *de novo* resistance to SN-38, by exposing the human BC cell lines MCF-7 and MDA-MB-231 to either stepwise increasing concentrations over 6 months or an initial high dose of SN-38 (the active metabolite of irinotecan), respectively. The resistant cell lines were analyzed for cross-resistance to other anti-cancer drugs, global gene expression, growth rates, *TOP1* and *TOP2A* gene copy numbers and protein expression, and inhibition of the breast cancer resistance protein (ABCG2/BCRP) drug efflux pump.

**Results:**

We found that the resistant cell lines showed 7–100 fold increased resistance to SN-38 but remained sensitive to docetaxel and the non-camptothecin Top1 inhibitor LMP400. The resistant cell lines were characterized by Top1 down-regulation, changed isoelectric points of Top1 and reduced growth rates. The gene and protein expression of ABCG2/BCRP was up-regulated in the resistant sub-lines and functional assays revealed BCRP as a key mediator of SN-38 resistance.

**Conclusions:**

Based on our preclinical results, we suggest analyzing the predictive value of the BCRP in breast cancer patients scheduled for irinotecan treatment. Moreover, LMP400 should be tested in a clinical setting in breast cancer patients with resistance to irinotecan.

**Electronic supplementary material:**

The online version of this article (doi:10.1186/s12885-016-2071-1) contains supplementary material, which is available to authorized users.

## Background

First-line chemotherapy of recurrent breast cancer (BC) is dependent on the type of prior adjuvant treatment but it most often consists of repeated cycles of anthracyclines and/or taxanes, possibly combined with cyclophosphamide, with standard combinations typically associated with response rates of about 50–60 % [1]. Second-line treatment may include 5-fluorouracil (5-FU), gemcitabine, or vinorelbine, and typically show response rates of 30–40 % [2]. Additional lines of treatment are also available in clinical management of BC, but whenever a new line of treatment is introduced patients show increasingly lower response rates. Evidently, there is a critical need for more efficient therapeutic intervention in metastatic breast cancer (mBC) since many BC patients are exposed to chemotherapy, with its ensuing side effects, without having any benefit from the treatment. An improved therapeutic index (benefit/side effect) may be achieved by identification of non-cross-resistant therapeutic options and/or by identification of relevant predictive biomarkers to be used to identify patients with the highest likelihood of benefit from a particular treatment.

Chemotherapeutic agents that target the Topoisomerase I protein (Top1) are routinely used in treatment of metastatic colorectal cancer [3] and have significant effect in other cancer types as well, including glioblastoma multiforme, upper gastrointestinal cancers, pancreatic cancer, ovarian cancer, small cell lung cancer and cervical cancer [4]. Currently used Top1 targeting drugs, such as irinotecan, etirinotecan (NKTR-102), or topotecan are derivatives of camptothecin. However, non-camptothecin derived next generation Top1 inhibitors, such as indenoisoquinolines, are currently being tested in clinical trials [4, 5] with promising results (http://clinicaltrials.gov/show/NCT01245192).

Irinotecan, etirinotecan and topotecan have also been tested in clinical trials in mBC [6], and irinotecan and etirinotecan regimens were shown to benefit a considerable proportion of mBC patients who had relapsed on prior treatment with anthracyclines and taxanes [7, 8]. The long-acting Top1 inhibitor etirinotecan has been evaluated in a randomized phase III study; the BEACON study [8, 9] . This study is based on data from a report that evaluated etirinotecan in 70 taxane-resistant mBC patients and an objective response rate of 29 % was observed when this drug was given as second- or third-line treatment [8]. These data was reproduced in the BEACON study, which also demonstrated that etirinotecan was at least as efficient as the physichian’s choice of treatment [9].

With expected objective response rates of approximately 25–30 % in pre-treated mBC patients, it is highly likely that many of the patients developed cross-resistance to Top1 inhibitors during their prior treatment. For example, Top1 and Top2 inhibitors, and taxanes are all known substrates for xenobiotic drug transporters from the ABC-cassette family, which may be up-regulated during chemotherapy treatment [10]. Alternatively, resistance to Top1 inhibitors may be pre-existing and could potentially also include resistance to anthracyclines and taxanes, e.g., through up-regulation of one or more common molecular drug resistance mechanisms even before exposure to treatment.

Irrespective of the pre-existence or induction of resistance, a validated molecular drug sensitivity/resistance profile might enable physicians to identify patients who are most likely to benefit from Top1 inhibitor treatment and only offer this therapy option to these patients. Such a strategy would increase the therapeutic index of Top1 inhibitors in mBC. In that respect, it has been shown that around 30 % of BC patients possess amplifications of the *TOP1* gene [11] and a clinical trial is currently investigating if increased *TOP1* gene copy numbers may be a predictive biomarker for response to irinotecan in BC patients [12]. However, no predictive biomarkers for Top1 inhibitor treatment are implemented for clinical use, meaning that the majority of mBC patients will experience drug-induced side effects without any therapeutic benefit. Moreover, there are unmet needs to establish exactly when Top1 inhibitors should be used in BC treatment, and to identify novel drug entities that are effective in irinotecan-resistant BC.

With the aims to search for Top1 inhibitor predictive molecular biomarkers and to identify which drugs are effective in irinotecan resistant BC cells, we have established human breast cancer cell line model systems for resistance to SN-38, the active metabolite of irinotecan. To cover a wide range of potential mechanisms, we have established four SN-38 resistant cell lines through exposure to either gradually increasing concentrations of SN-38 or a single high dose. We describe here potential molecular mechanisms of SN-38 resistance in breast cancer cells and sensitivity to other commonly used chemotherapeutic agents, as well as novel non-camptothecin Top1 targeting drugs.

## Methods

### Chemicals and drugs

SN-38 and Ko143 (Sigma-Aldrich, Copenhagen, Denmark), Epirubicin (2 mg/ml, Actavis Nordic A/S, Gentofte, Denmark), Cisplatin (1 mg/ml, Hospira, Denmark) and Docetaxel (20 mg/ml, Actavis Nordic A/S, Gentofte, Denmark). The indenoisoquinoline drugs LMP776 (NSC 725776) and LMP400 (NSC 743400) [4, 5, 13], were provided by the NCI Developmental Therapeutics Program (DTP), National Cancer Institute, NIH, Bethesda, MD, USA. All drugs were dissolved in dimethyl sulfoxide (DMSO) and stored at −20 °C. Drugs were dissolved in culture medium immediately before use.

### Cell cultures

A panel of the 52 breast cancer cell lines was propagated in complete media: RPMI 1640 (Gibco, Invitrogen, Denmark) with 10 % fetal bovine serum (FBS, Gibco, Invitrogen, Denmark) as previously described [14]. Docetaxel resistant MCF-7 and MDA-MB-231 breast cancer cell lines were grown as previously described [15].

### Establishment of SN-38 resistant human breast cancer cell lines

The human breast cancer cell line MDA-MB-231 was obtained from American Type Culture Collection (ATCC, Rockville, MD). Professor Ole William Petersen (University of Copenhagen) kindly provided the human MCF-7 breast cancer cell line. MCF-7 and MDA-MB-231 cell lines were maintained in DMEM including L-glutamine medium (Gibco, Life Technologies, USA) supplemented with 10 % FBS (Gibco Invitrogen, USA) for MDA-MB-231 cells and 5 % FBS, 1 % non-essential amino acids (NEAA, Life Technologies, USA) for MCF-7 cells. Cell lines were cultured in the presence of penicillin/streptomycin antibiotics (100 U/mL, Invitrogen) and incubated at 37 °C in a humidified environment containing 5 % CO_2_. SN-38-resistant and DMSO-exposed control cell lines were maintained under the same medium conditions, supplemented with SN-38 or DMSO, respectively. All experiments were carried out in presence of SN-38 except for growth curves, cell cycle analysis and Top1 enzyme activity. The resistant cell lines were designated “acq” for acquired resistance and “de novo” for *de novo* resistance.

Initially, IC_50_ values for SN-38 were determined by exposing cell lines to a range of SN-38 concentrations using methylthiazolyldiphenyl-tetrazolium bromide (MTT) assays to measure the response. To establish acquired resistance, the parental cell lines were exposed to SN-38 concentrations ranging from 5000- to 500-fold lower than IC_50_ and the highest concentration that only caused minimal effect on the cells was chosen as a starting point. The cell lines with acquired resistance, MDA_acq_ and MCF-7_acq_, were developed by exposing the parental cell lines to stepwise increasing concentrations of SN-38 (from 3.6nM – 68.2nM for MDA_acq_ and 3.0nM – 36nM for MCF-7_acq_) over 6 months. SN-38 exposed cell lines were maintained at each drug concentration for three passages. Two resistant cell lines were obtained after a total of 24 passages for MDA_acq_ and 23 passages for MCF-7_acq_ in presence of SN-38. To establish the de novo resistant cell lines we initially exposed parental cell lines to SN-38 concentrations ranging from the low nM range up to 25 μM and selected the cell populations that eventually were able to re-grow with constant exposure to SN-38. Thereby, we selected de novo resistant cell lines, MDA_de__novo_ and MCF-7_de__novo_, which survived constant exposure to 24nM for MDA_de__novo_ and 12nM for MCF-7_de__novo_ (Table 1). The identity of both parental and resistant cell lines was confirmed by Short Tandem Repeat (STR) analysis (Identi Cell, Aarhus, Denmark). In addition, all cell lines were confirmed to be mycoplasma-free (Mycoplasma PCR Detection Kit, Minerva Biolabs, Berlin, Germany).

**Table 1:** Establishment of SN-38 resistant cell lines

Cell Lines	SN-38 Dose	Final SN-38 concentration	IC_50_ Values (μM)		RR
			Resistant cell lines	DMSO control	
MDA_acq_	Stepwise (3.6nM-68.2nM)	68.2nM	40.2 ± 4.0	5.6 ± 0.9	7.2
MCF-7_acq_	Stepwise (3.0nM-36nM)	36nM	33.6 ± 3.0	3.7 ± 0.5	9.1
MDA_de novo_	Constant (24nM)	24nM	66.8 ± 16.2	0.7 ± 0.5	95.4
MCF-7_de novo_	Constant (12nM)	12nM	31.9 ± 0.6	2.3 ± 0.9	13.9

Mean IC_50_-value (μM) ± standard deviation of three independent experiments. RR; relative resistance is the IC_50_-value of the resistant cell line divided by the IC_50_-value of their corresponding DMSO controls

### Data mining of publicly available datasets

We extracted the IC_50_ values reported for topotecan [16] and camptothecin [17] for the breast cancer cell lines present in our panel of 52 cell lines and correlated the IC_50_ values to the *TOP1* copy numbers.

### Cytotoxicity assay

In vitro drug resistance and cross-resistance were determined using the MTT and crystal violet assays as previously described [15]. Cell lines were plated for 48 h and then exposed to drugs for 72 h. Using GraphPad Prism, IC_50_ values of three independent repeats were calculated to determine the change in resistance.

### Cell growth and doubling time analysis

For the growth assay, 40,000 cells/well for MDA-MB-321 and 60,000 cells/well for MCF-7 were seeded in 6-well plates. Cells were harvested at 24 h intervals for days 1 to 8. The assay was conducted once and each well was counted manually three times at each interval. Average values were used to plot the growth curves. The doubling time of the cells in exponential phase was calculated using the formula: Doubling time = h ∗ ln(2)/ln(c2/c1), where c1 and c2 represent the cell numbers at the beginning and end of the exponential phase during time (h), respectively [18].

### Cell cycle analysis by FACS

Fixation, propidium iodide staining and cell cycle analysis using fluorescence-activated cell sorting (FACS, BD FACSVerseTM) and data analyses by FlowJo software were done as previously described [19] and analyses were repeated in two independent replicates.

### Formalin fixation and paraffin embedding of cells

Cells were formalin fixed, embedded in agarose and paraffin embedded as previously described [20].

### FISH analysis

A *TOP1*/CEN-20 probe mix [20] and *TOP2A* FISH pharmDx™ kit (Dako, Denmark, #K5333) were applied according the manufactures instructions as previously described [21]. This analysis was conducted once.

### Copy number data and mRNA microarray analysis on the 52 breast cancer cell lines

Gene expression dat obtained by Affymetrix U133 microarray (GEO entry GSE41313) were applied [14]. Copy number data were generated using SNP6 chips from Affymetrix (Santa Clara, USA). Raw data were pre-processed using Nexus software (BioDiscovery, Hawthorne, USA) using the recommended settings for SNP calling, segmentation and copy number status provided by Nexus software.

### mRNA microarray analysis on parental and the acquired resistant cell lines

Total RNA was extracted from three independent passages of 70 % confluent cells for each of the four cell lines (MCF-7 parental, MCF-7_acq_, MDA-MB-231 parental and MDA_acq_) using Trizol reagent (Invitrogen). Expression analysis using Agilent Human Gene Expression Microarrays (G4845A, Agilent Techologies, Santa Clara, CA, USA), image quality, background correction and normalization were conducted as previously described [22]. Sample clustering was performed by the ‘ward’ method in the software R (http://www.r-project.org/). Statistical tests were performed using a moderated *t*-test, and p-values were adjusted for multiple testing by the Benjamin & Hochberg method [23]. Genes were considered significantly differentially expressed if the adjusted p-value < 0.05 and the absolute log2-fold change > 0.8. Gene Set Enrichment Analysis (GSEA) was performed using the Clusterprofiler package [24] using Reactome, KEGG and the GeneOntology data distributed Bioconductor project. Network analysis was performed using MetaCore from Thomson Reuters. Networks were constructed based on direct interactions in the MetaCore database for all deregulated genes with a log2 fold change > 1 and *p*-value < 0.05. For each cell line three biological replicates were analyzed.

### Availability of data and materials

The gene expression dataset supporting the conclusions of this article is available in the ArrayExpress repository, accession number E-MTAB-3224, https://www.ebi.ac.uk/arrayexpress/experiments/E-MTAB-3224.

### Protein purification, western blotting and Peggy analyses

Cells were grown to 70 % confluence and western blotting was performed as previously described [22]. Primary antibodies were incubated at 4 °C overnight (Top1 (Abcam, UK, 1:2000), Top2A (OriGene Technologies, 1:500), BCRP (Abcam, 1:1000), MDR1 (Novus biological. Denmark, 1:1000) and β-actin (Sigma-Aldrich, Denmark, 1:15,000,000)). Species-specific horseradish peroxidase-labeled secondary antibodies were applied for 1 h at 37 °C (Anti-rabbit (Dako, Denmark): Top1 (1:10,000): and anti-mouse (Dako, Denmark): BCRP (1:4000), MDR1 (1:5000), β –actin (1:5000)). Protein bands were quantified using ImageJ software. Three independent biological replicates were analyzed for each cell line.

Peggy analysis was performed on a nanocapillary electrophoresis analysis system (ProteinSimple, USA) for size, amount and pI pattern of Top1 (28). For size analysis, cell lysates were diluted in MPER lysis buffer with Bicine/CHAPS lysis buffer containing 2 % DMSO inhibitors (ProteinSimple) and 4X master mix/fluorescent standards (Protein Simple) according to the manufacturer’s instructions. The samples were denatured for 10 min at 70 °C and pipetted into a 384-well plate along with primary antibodies (Top1 (Abcam) 1:100, β-actin (Abcam) 1:100) diluted in Antibody Diluent Plus (ProteinSimple) according to the manufacturer’s instructions. For charge analysis, lysates were prepared in Bicine/CHAPS lysis buffer containing 2 % DMSO inhibitors and diluted with this and premix G2 pH 3–10 separation gradient (ProteinSimple) containing 2.2 % ladder 1 (ProteinSimple). Charge analysis samples were pipetted into a 384-well plate along with primary antibodies (Top1 (Abcam) 1:50, β-actin (Abcam) 1:50) diluted in antibody diluent (ProteinSimple) according to manufacturer’s instructions. Data were processed using Compass software (ProteinSimple). The size analyses were repeated four times with two independent biological replicates and the charge analyses were conducted in biological triplicates.

### Top1 enzyme activity assay

The cellular Top1 enzyme activity was analyzed as previously described [25]. Briefly, cell lines were grown in the absence of SN-38, trypsinized, counted, centrifuged and 1 million cells were pelleted for analyses in the presence or absence of SN-38. Activity assays were conducted in triplicate with three independent biological replicates.

### BCRP drug-efflux pump inhibition

Cells were seeded at 10,000 cells/well in 96-well plates and allowed to adhere for 48 h at 37 °C. Cell lines were exposed to SN-38 with or without Ko143, a specific BCRP inhibitor (Sigma Aldrich) for 72 h. Cell viability was assessed using MTT assays with triplicate determinations from three independent biological passages.

### Statistics

MTT data were analyzed in Excel by two-tailed Student’s *T*-test assuming equal variance and p-values below 0.05 was considered statistically significant. A non-parametric test (Kruskall-Wallis) was used to associate the *TOP1* copy number (CN)-status with mRNA expression levels with the order of groups being CN loss, CN neutral and CN gain and a two-sided p-value below 0.05 was considered statistically significant.

## Results

### Topoisomerase 1 as predictor of response to SN-38

Increased *TOP1* CN, Top1 mRNA or protein expression or enzyme activity has been correlated to cancer cells’ sensitivity to Top1 targeting drugs [20, 26–29]. To further explore this in breast cancer cells, we initially applied a FISH *TOP1*/CEN-20 probe mixture [20] to a repository consisting of 52 human breast cancer cell lines to analyze the distribution of *TOP1* and CEN-20 CN and the *TOP1*/CEN-20 ratios in these cell lines (Additional file 1: Figure S1a). The *TOP1* CN varied from 1.2 (BT474) to 5.5 (HCC1419). There was a trend towards association between *TOP1* and CEN-20 copy numbers as reflected in a *TOP1*/CEN-20 ratio close to 1 for the majority of the cell lines (Additional file 1: Figure S1a). Comparing FISH-derived *TOP1* CNs to either SNP-derived CNs or to *TOP1* mRNA expression we found significant associations (*p* < 0.0001 and *p* = 0.0012, respectively) indicating that the *TOP1* genes are actively transcribed even in cell lines with many *TOP1* copies (Additional file 1: Figure S1b,c). Based on the status of *TOP1* CN, HER2 and estrogen receptors (ER), we then selected 9 breast cancer cell lines from the panel of the 52 characterized cell lines (Additional file 1: Figure S1d). In these selected cell lines the Spearman’s correlation coefficient between the *TOP1* CN and gene expression levels was 0.64 (*p* = 0.067) and Top1 appeared to be functional because the cellular Top1 enzyme activity could be impaired by SN-38 (Additional file 1: Figure S2b). The *TOP1* CN corresponded well to the Top1 protein levels observed in western blots, except for the HCC70 cell line (Additional file 1: Figure S2a), and also correlated significantly (*r* = 0,70, *p* = 0.035) to the Top1 enzyme activity (data not shown). These 9 cell lines were then tested for sensitivity to SN-38 (Additional file 1: Figure S2c) to evaluate the correlation of *TOP1* CN or *TOP1* gene expression and sensitivity to SN-38. We found non-significant negative Spearman’s correlation coefficients between the IC_50_ values and the *TOP1* CN (*r* = −0.20, *p* = 0.61) or the *TOP1* mRNA (*r* = −0.17, *p* = 0.64). However, the Spearman’s correlation coefficients between the Top1 activity and the *TOP1* CN or the *TOP1* mRNA were (*r* = 0.65, *p* = 0.067) or (*r* = 0.95, *p* = 0.0004), respectively. This indicates that more *TOP1* copies and higher Top1 enzyme activity correlate to increased sensitivity to SN-38.

We mined publicly available datasets to explore the correlation between our *TOP1* CN data to IC_50_ values reported for topotecan [16] and camptothecin [17] in breast cancer cell lines. In agreement with our data, the *TOP1* CN had non-significant negative Spearman’s correlations to the IC_50_ values for topotecan (*r* = −0.37, *p* = 0.087) and for camptothecin (*r* = −0.24, *p* = 0.38). Thus, factors beyond *TOP1* CN, Top1 expression and enzyme activity appear to be involved in the response to SN-38. Based on the results presented above we selected two cell lines to represent the majority of BC patients likely to be candidates for Top1 targeted therapy, namely the MDA-MB-231 and the MCF-7 cell lines, which represent cell lines with *TOP1* CN gain (Additional file 1: Figure S1a,d) and estrogen receptor/HER2 negative or positive cells, respectively.

### Establishment and characterization of resistant cell lines

**Fig. 1:**
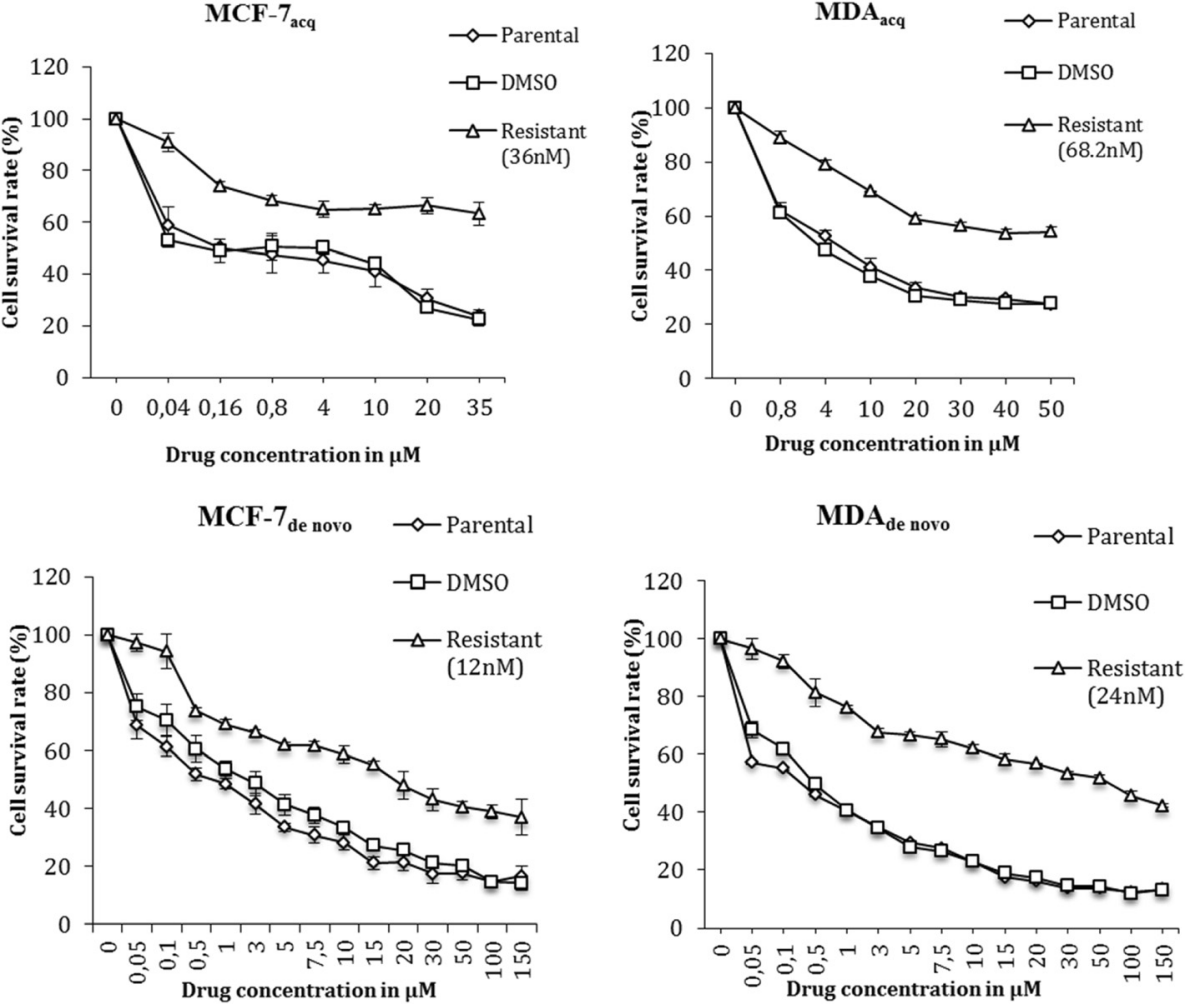
Sensitivity to SN-38 in the established SN-38 resistant cell lines in comparison to their controls. Using MTT assay, cells were exposed to the shown SN-38 concentrations for 72 h. Triplicate wells were analyzed, and data shown is mean ± s.d. of a representative experiment in percentage. *n* = 3

The resistant model systems were established as described in Materials and Methods (Table 1) and the resistant phenotypes of the established cell lines were initially confirmed by exposing the cell lines to their final SN-38 concentration (68.2nM, 36nM, 24nM and 12nM, respectively, Additional file 1: Figure S3). These MTT data confirmed significant increase in resistance to SN-38 and the MDA_acq_ MCF-7_acq_ and MDA_de novo_ and MCF-7_de novo_ cell lines were found to be 7, 9, 95 and 14-fold more resistant to SN-38, respectively, when comparing the IC_50_ to their corresponding parental and DMSO controls (Fig. 1, Table 1). Similar results were shown by crystal violet assay (data not shown). The resistant phenotype was stable for all cell lines as the resistance to SN-38 was retained after withdrawal of SN-38 containing media for 1 month (data not shown).

### Growth curves

The growth curves (Additional file 1: Figure S4) illustrate that the resistant cell lines had significantly (*p* < 0.05) lower growth rates when compared to their parental and DMSO controls (all cell lines were grown in the absence of SN-38). The doubling times of MDA_acq,_ MCF-7_acq,_ MDA_de novo_ and MCF-7_de novo_ were 35.5, 44.3, 43.9 and 33.2 h, respectively, which was 7.8, 11.7, 16.8 and 6.1 h longer than their respective DMSO controls. The increase in doubling times was significant (*p* < 0.05) for all cell lines except for MCF-7_de novo_ (*p* = 0.08).

### Cell-cycle distribution

Cell cycle analyses were performed without adding SN-38 to investigate the reason for longer doubling time of the resistant cell lines, and the results showed that the resistant cell lines had increased percentage of cells in G2/M phase and decreased percentage of cells in G0/G1 phase in comparison to their parental controls (Additional file 1: Figure S5), which indicated cell cycle arrest at the G2/M phase as a factor for increased doubling time of the resistant cell lines.

### Cross-resistance to other anti-cancer drugs

The SN-38 resistant cell lines were assessed for cross-resistance to a range of anticancer drugs (cisplatin, docetaxel, epirubicin, LMP400 and LMP776) in comparison to their DMSO controls. Resistant cell lines exhibited different and complex patterns of cross-resistance to various anti-cancer drugs, which is summarized in Table 2. Most strikingly, a consistent pattern was observed with docetaxel as none of the SN-38 resistant cell lines had developed cross-resistance to this drug. Interestingly, the docetaxel resistant MCF-7 and MDA-MB-231 cell lines were cross-resistant to SN-38 (Additional file 1: Figure S6). For epirubicin, both MDA_acq_ and MDA_de novo_ showed cross-resistance whereas the MCF-7_acq_ and MCF-7_de__novo_ remained sensitive. For the indenoisoquinoline Top1-targeting drug LMP776, which is a weak substrate for BCRP, three out of four cell lines showed cross-resistance, whereas three out of four SN-38 resistant cell lines remained sensitive to LMP400 (NSC 724998), which is not a BCRP substrate [5]. For cisplatin, the cross-resistance pattern was complex and two out of four cell lines demonstrated cross-resistance (Table 2).

**Table 2:** Drug sensitivity IC_50_-values and Relative resistance

Anti- cancer drugs	Epirubicin	RR	Docetaxel	RR	Cisplatin	RR	LMP776	RR	LMP400	RR
MDA_acq_ DMSO	0.4 ± 0.2	4.3	22.0 ± 9.9	1.0	62.8 ± 13.7	1.1	8.9 ± 11.7	5.0	10.5 ± 9.7	1.2
MDA_acq_	1.7 ± 0.3		21.0 ± 5.3		70.6 ± 4.1		44.6 ± 45.7		12.4 ± 12.2	
MCF-7_acq_ DMSO	0.9 ± 0.5	1.9	20.4 ± 2.3	1.3	26.1 ± 9.5	2.5	^a^		^a^	
MCF-7_acq_	1.7 ± 0.9		25.8 ± 8.2		65.6 ± 53.4		^a^		^a^	
MDA_de novo_ DMSO	5.2 ± 3.2	3.4	18.0 ± 3.7	1.5	34.7 ± 7.03	2.7	11.0 ± 6.2	2.0	15.8 ± 6.3	1.6
MDA_de novo_	17.6 ± 1.4		27.3 ± 2.1		95.1 ± 4.9		22.5 ± 4.3		25.8 ± 3.5	
MCF-7_de novo_ DMSO	8.1 ± 9.1	1.2	24 ± 7.5	1.5	45.6 ± 5.4	1.2	40.0 ± 14.2	1.4	42.6 ± 36.3	1.4
MCF-7_de novo_	10.0 ± 7.9		34.9 ± 10.2		53.7 ± 13.0		55.9 ± 28.6		58.2 ± 36.5	

Mean IC_50_-value (μM) ± standard deviation of three independent experiments. RR; relative resistance is the IC_50_-value of the resistant cell line divided by the IC_50_-value of their corresponding DMSO controls ^a^Cross-resistance by looking at the graphs as 50 % inhibition was not achieved with these drugs

### Florescence in-situ hybridization (FISH)

To investigate whether the acquired resistance to SN-38 correlated to aberrations in *TOP1* or *TOP2A* at the gene level, FISH analysis was performed on parental, MDA_acq_ and MCF-7_acq_ cell lines. No differences in the *TOP1* or *TOP2A* CN between the resistant cell lines and the respective control cell lines were detected. Furthermore, neither *TOP1*/CEN-20 nor *TOP2A*/CEN-17 ratio numbers were different among the respective cell lines (Additional file 1: Table S1).

### Gene expression analysis

**Fig. 2:**
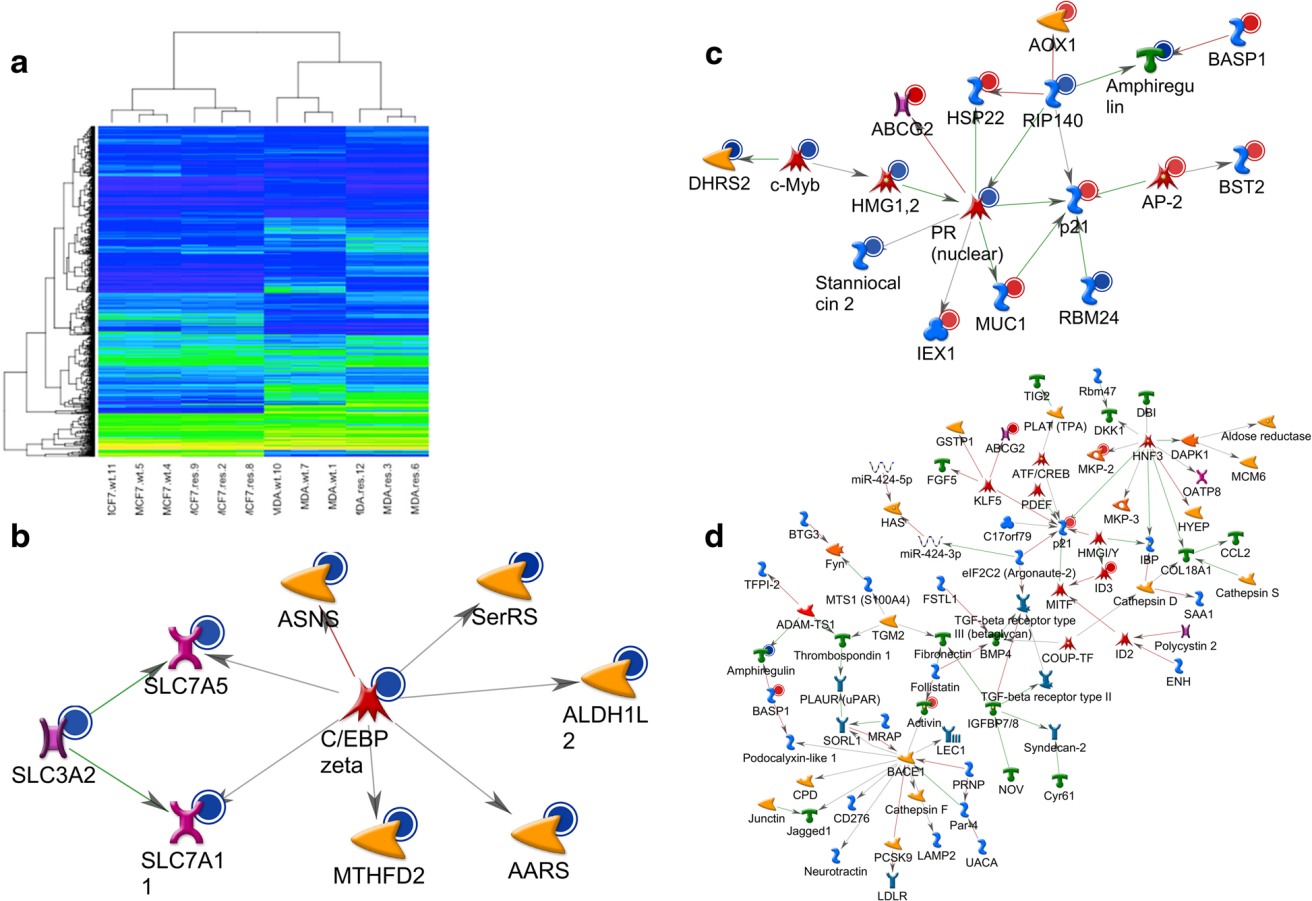
Global expression analysis of resistant (res) and non-resistant DMSO control cell lines (wild type, wt). **a** Heatmap showing differentially expressed genes (see text), the rows are genes, columns samples and the colors show the normalized expression level, yellow being high expression and blue low. **b, c, d** MetaCore analyses of networks among genes differentially expressed in the resistant cells. In MCF-7_acq_ two networks were identified (**b**, **c**) and in the MDA_acq_ a single netwwotk was indetified (**d**). Network formation was established based on known direct interactions

Genome-wide gene expression analyses on the DMSO control and the two SN-38 acquired resistant cell lines identified differentially expressed genes, which are visualized with a heatmap (Fig. 2a). The MCF-7 cells and MDA-MB-231 cell lines clustered separately; however, resistant cell lines did not cluster separately from DMSO controls (Additional file 1: Figure S7b). The MDA_acq_ model system had numerous differentially expressed genes, 32 of these genes being differentially expressed in common to both the MDA and the MCF model systems (Additional file 1: Figure S7b and Additional file 2).

The ABCG2 transcript, encoding the breast cancer resistance protein (BCRP), was the most up-regulated in the MDA_acq_ system (32 fold up-regulation) and the second most deregulated in the MCF-7_acq_ system (4 fold up-regulation) (Additional files 3 and 4). Only one other gene in the top 50 was deregulated in common, namely ID3 (inhibitor of DNA binding 3), which was 4 fold up-regulated in both MDA_acq_ and MCF-7_acq_. It is of interest to note that the ABCB1 transcript, encoding the permeability-glycoprotein (Pgp/MDR1) was not deregulated in the acquired resistance cell lines. Gene Ontology (GO) Molecular Function analysis found that the most prominent common GO term was the category “transcription cofactor activity”, defined by genes that interact selectively and non-covalently with a regulatory transcription factor and also with the basal transcription machinery in order to modulate transcription. Reactome and KEGG pathway analyses identified among the top 10 pathways “Metabolism of xenobiotics by cytochrome P450”, “Nucleosome assembly” and “DNA damage/Telomere stress induced senescence” (data not shown). In the MCF-7_acq_ the MetaCore network analysis of the deregulated genes highlighted two networks of 19 and 10 genes with the most connecting nodes being the transcription factors PR and FUS/DDIT3, the kinase inhibitor p21 and the regulatory protein RIP140 (Fig. 2b,c). In the MDAacq cells a network of 94 genes was highlighted and the most connecting nodes being the transcription factors HNF3 and PAX8, as well as the protease BACE1 and the kinase inhibitor p21 (Fig. 2d). These nodes might represent important causative changes for the development of resistance in the model system.

### Differential protein expression in SN-38 resistant cell lines

**Fig. 3:**
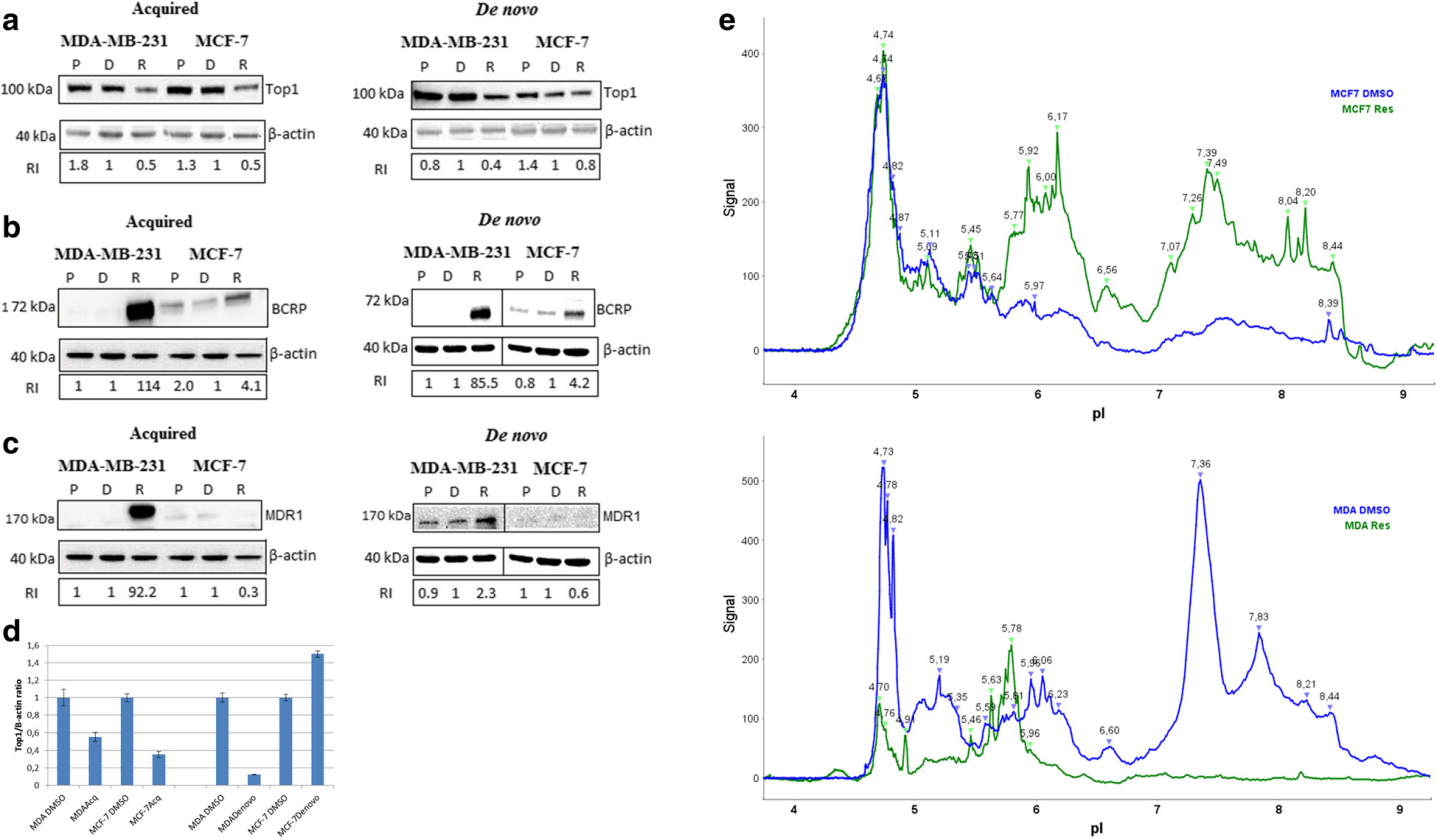
Western blotting of total protein extracts from SN-38 resistant (R) in comparison to their respective controls (Parental (P) and DMSO (D)). All samples were immunoblotted with an antibody to β-actin to illustrate equal protein loading (**a**): Western blotting of Top1/β-actin; (**b**): BCRP/β-actin; (**c**): MDR1/β-actin. Protein bands were quantified by image J software. Number represents relative intensity (RI) bands to their respective DMSO control. **d** Top1 and β-actin proteins signals from nanocapillary electrophoresis were quantified and expressed in percent of the relevant DMSO control cell lines. All differences were signicant (*p* < 0.05) with *p*-values of 0.0088, 0.00012, 0.0017 and 0.00018 for MDA_acq_, MCF-7_acq_, MDA_de novo_ and MCF-7_de novo_, respectively. **e** Visualization of the Top1 isoelectric patterns in the cell lines MCF-7acqDMSO, MCF-7acq (*top panel*) and MDAacqDMSO, MDAacq (*lower panel*)

The expression of proteins previously suggested to be involved in resistance to SN-38 (Top1, Top2a, BCRP and MDR1) was evaluated by Western blotting (Fig. 3 and Additional file 1: Figure S8). The levels of Top1 were markedly reduced (<50 %) in MDA_acq,_ MCF-7_acq_ and MDA_de novo_ cell lines while only a minor decrease in Top1 protein expression was seen in MCF-7_de novo_. Top2a levels remained unchanged in all resistant cell lines (Additional file 1: Figure S8). A clearly increased protein expression of the drug efflux pumps MDR1 and BCRP was observed in the MDA_acq_ cell line. Likewise, MDA_de novo_ had a strong BCRP over- expression; however, it had only a minor increase in MDR1 expression. A minor increase in expression of BCRP was observed in MCF-7_acq and_ MCF-7_de novo_ while no expression of MDR1 was detected in any of the MCF-7 cell lines_._

We applied nanocapillary electrophoresis for more precise quantification of the Top1 protein. Top1 and β-actin were simultaneously detected and fully separated without background signals (Additional file 1: Figure S9). The Top1 signals were normalized by the β-actin signals and compared between resistant and DMSO control cell lines (Fig. 3d). This showed significant (*p* < 0.05) down regulation of Top1 in MDA_acq_ (0.55x), MCF-7_acq_ (0.35x) and MDA_de novo_ (0.12x) whereas the MCF-7_de novo_ showed a slight increase (1.5x). Post translational modifications (PTMs) of Top1 have been associated with sensitivity to Top1 targeting drugs [18, 30] and we therefore analyzed the Top1 isoelectric patterns in the cell lines (Fig. 3e). Overall, the Top1 pI charge profiles were alike in the MCF-7 and MDA-MB-231 DMSO controls with prominent peak signals in the area around 5.5 and 7.5-8.5. When MCF-7_acq_ cells were compared to MCF-7 control cells the pI peak pattern was very similar to the MCF-7 DMSO controls indicating no major changes in Top1 PTMs in the resistant MCF-7 cells. However, the MDA_acq_ cells lost the peak signals in the pI range around 5.5 and 7.5-8.5 but gained peak signals around pI 5 and pI 6.4. These data indicates that Top1 PTMs are changed in the MDA_acq_ cells.

### Inhibition of BCRP drug-efflux pump

**Fig. 4:**
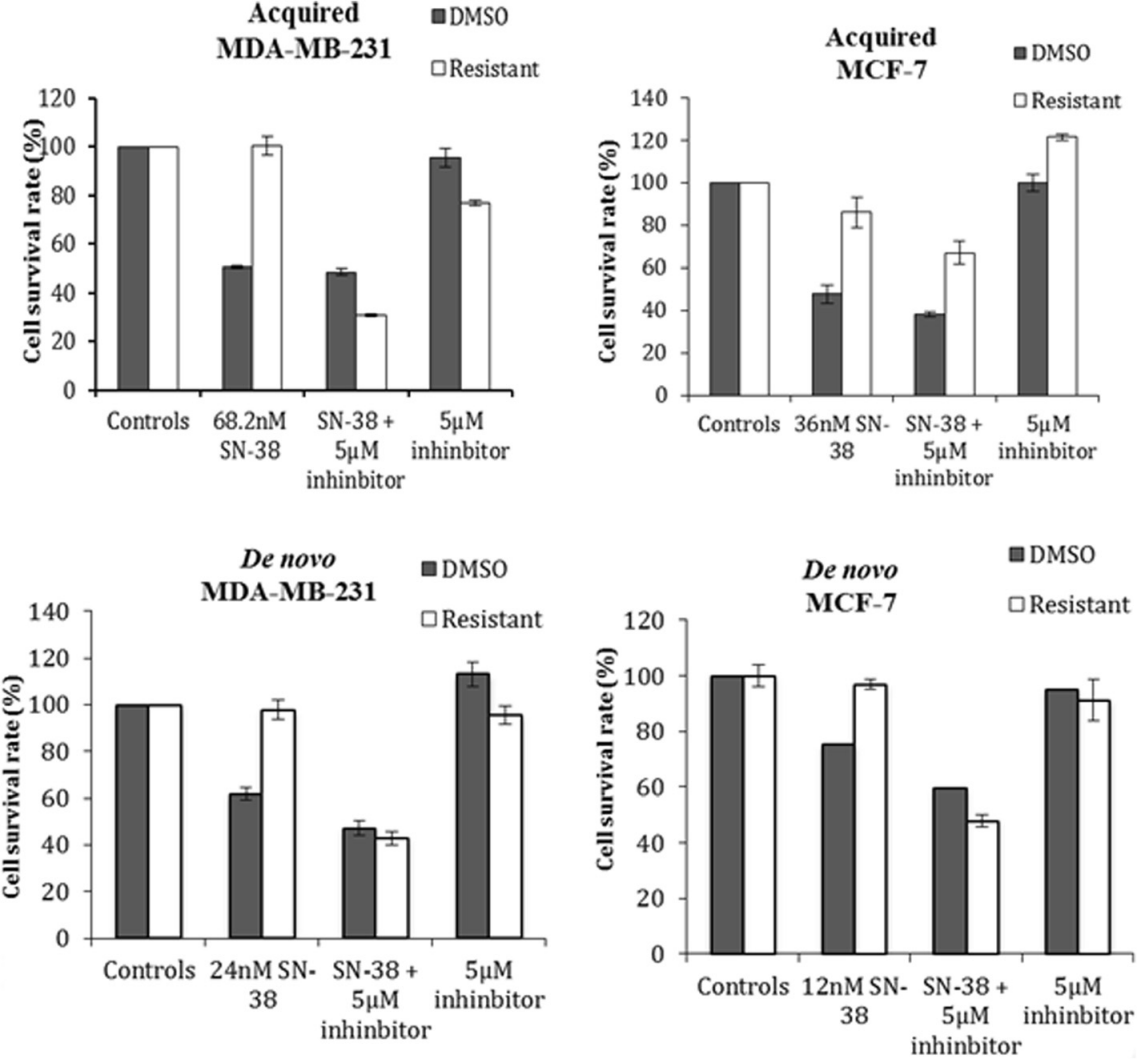
Inhibition of the BCRP efflux pump in SN-38 resistant cell lines in comparison to their controls in MTT assay. Cells were exposed to their corresponding final SN-38 concentration with or without Ko143 (5 μM) for 72 h. Triplicate wells were analyzed, and data shown is mean ± s.d. of a representative experiment in percent of untreated cells (controls), *n* = 3. The *p*-values between SN-38 treated and SN-38 + 5 μM inhibitor were all below 0.05 (the *p*-values for MDA_acq_, MCF-7_acq_, MDA_de novo_ and MCF-7_de novo_ were 9.6E-5, 0.01, 7.2E-5 and 0.0001, respectively)

To investigate the functional importance of the observed up-regulation of the BCRP pump in SN-38 resistance in breast cancer cells, we inhibited its activity using Ko143, a specific inhibitor of this pump [31, 32]. The Ko143 compound did not have any effect on cell survival and did not interfere with the effect of SN-38 on the DMSO control cell lines. Co-treatment with Ko143 and SN-38 resulted in significant (*p* < 0.05) and complete re-sensitization of the MDA_acq_, MDA_de novo_ and MCF-7_de novo_ cell lines to the level of DMSO control cell line. Although significant (*p* = 0.01) the MCF-7_acq_ was only slightly re-sensitized to SN-38 following Ko143 co-treatment (Fig. 4).

## Discussion

Resistance to treatment is a major obstacle in the current management of BC patients. As response rates to Top1 inhibitors in mBC is around 30 % [6, 8] it is clear that the majority of patienst are or become resistant. In order to develop novel non-cross-resistant agents and to identify predictive molecular markers, a more detailed insight into the underlying molecular mechanisms of drug resistance is necessary. Although the occurrence of resistance to camptothecins is not understood in detail, it is thought to be mediated by multiple mechanisms, including, but not limited to, reduced drug-target interactions, down-regulation of the Top1 enzyme, *TOP1* gene mutations and increased drug efflux, resulting from up-regulated expression of efflux drug transporters [4, 33, 34]. We report here the first preclinical model system of SN-38 drug resistance in BC. After testing 52 human BC cell lines, we selected two lines based on SN-38 sensitivity, *TOP1* gene copy status, HER2 status and estrogen receptor protein expression, and used these two cell lines to develop SN-38 resistance by either exposing them to stepwise increasing concentrations of SN-38 over a period of 6 months or by selecting the cell population that survived exposure to the highest SN-38 concentration.

Some common traits were identified in the SN-38 resistance mechanisms of all four SN-38 resistant cell lines, independently of these cell lines being de novo resistant or having acquired resistance, and also irrespectively of whether or not the cell lines expressed estrogen receptor (Table 2 and Figs. 3 and 4). Two of these were particularly evocative: BCRP/ABCG2 and ID3.

### BCRP

We found mRNA and protein up-regulation of the xenobiotic drug transporter BCRP in the SN-38 resistant BC lines. BCRP has previously been shown to transport camptothecins [35] and to confer resistance to SN-38 in colon cancer cell lines [5, 36] and non-small cell lung cancer cells [37]. We found that BCRP was functionally involved in resistance to SN-38 in our BC lines as inhibition of BCRP reverted the resistance phenotype of the SN-38 resistant BC cell lines, although the resistant phenotype of MCF-7acq was only slightly re-sensitized (Fig. 4). Thus, development of resistance may be multifactorial and clonal bias may also contribute to differences in the specific molecular mechanisms for resistance to SN-38. These data are consistent with results obtained with a mouse model of BC that identified over-expression of BCRP as an in vivo mechanism of resistance to topotecan and showed that genetic ablation of BCRP increases survival of topotecan treated animals [38]. Similarly, in a BCRP (−/−) mouse model with orthotopically transplanted BC tumor cells, co-treatment with topotecan and the BCRP inhibitor Ko143 significantly increased survival compared to topotecan monotheraphy [32]. These pre-clinical data and the fact that BCRP is highly expressed in aggressive breast cancer subtypes [39, 40] makes this drug efflux transporter interesting as predictive biomarker and/or as a therapeutic target.

### ID3

ID proteins are helix-loop-helix (HLH) proteins that can form heterodimers with other HLH proteins and inhibit its interaction partner in binding to DNA. ID3 is involved in tumor growth, invasiveness, metastasis, and angiogenesis [41], and was up-regulated in the SN-38 resistant BC cell lines (Additional file 1: Figure S10). This is the first report to associate ID3 with resistance to SN-38 and one possible explanation for this observation is that negative regulation of DNA-binding by dimer partners slows down cell cycle and results in increased numbers of cells in the G0/G1 phase - as we found in our cell cycle analyses- which in turn will reduce sensitivity to SN-38 (Additional file 1: Figure S5).

Taxanes are currently regarded as the most efficient drug class in the management of BC and are commonly used in neoadjuvant and adjuvant therapy of BC and in management of mCB. In spite of showing overall response rates of more than 40 %, patients progress in a relative short time, and in many cases show cross-resistance to second-line anthracyclines and subsequent regimens. Interestingly, none of the SN-38 resistant cell lines had acquired cross-resistance to docetaxel while both of the MDA-MB-231 SN-38 resistant cell lines displayed minor cross-resistance to the anthracycline epirubicin (Table 2). It is quite intriguing that even in the MDA_acq_ cell line, which had a very strong up-regulation of the MDR1 protein, the cells retained sensitivity to docetaxel. Furthermore, our previously reported docetaxel resistant BC cell lines showed cross-resistance to SN-38 (Additional file 1: Figure S6). With an objective response rate of only 30 % to irinotecan in docetaxel-refractory mBC, the present preclinical results indicate that irinotecan could be administered prior to docetaxel to minimize cross-resistance between these two drugs. Although epirubicin is not considered a substrate for BCRP, a recent study has shown that high expression of BCRP correlated with resistance to epirubicin in colorectal cancer cells [42] and this is consistent with our findings in the SN-38 resistant MDA-MB-231 cell lines with high BCRP expression. To try to address the issue of cros-resistance, we included two novel indenoisoquinoline Top-1 inhibitors, LMP400 and LMP776, in this study, both of which are in early clinical trials and are well-tolerated (https://clinicaltrials.gov/ct2/show/NCT01051635, https://clinicaltrials.gov/ct2/show/NCT01794104). Remarkably, LMP400 did not display cross-resistance with SN-38 in three of the four SN-38 resistant BC cell lines (Table 2), which may be explained by the fact that LMP400 (NSC 724998) is not a substrate for BCRP [5]. This suggests a therapeutic potential for LMP400 in BC following disease recurrence upon irinotecan treatment. In contrast, 3 of the 4 SN-38 resistant cell lines were cross-resistant to LMP776 (Table 2).

Clearly, factors beyond ABCG2/BCRP may be involved in resistance to SN-38. To address other possibilities we explored other, known, molecular mechanisms underlying resistance to SN-38. In a panel of breast cancer cell lines with varying levels of *TOP1* CN, *TOP1* mRNA, Top1 protein and enzyme activity, we found consistency between the *TOP1* CN and protein levels (Additional file 1: Figure S2). Furthermore, higher *TOP1* mRNA levels were significantly associated with increased enzyme activity and non-significantly associated to increased sensitivity to SN-38, which is supported by other studies using fewer cell lines [20, 26–29, 43, 44]. The level of Top1 protein was reduced in the MDA_acq_, MCF-7_acq_ and MDA_de novo_ cell lines whereas only a minor decrease was observed in the MCF-7_de novo_. These results recapitulate data from a BC mouse model of acquired topotecan resistance that suggested reduced levels of Top1 as a mechanism of in vivo resistance [38]. The mechanisms causing reduced levels of Top1 protein remain elusive as no changes were observed in the *TOP1* CN or gene expression. However, this suggests that acquired resistance to SN-38 might involve a decrease in Top1 protein levels, which is also supported by studies demonstrating a correlation between reduced Top1 levels and reduced sensitivity to Top1 targeting drugs [45, 46]. The most likely reason for these observations is that lower levels of Top1 leads to reduction in the covalent-complex-dependent double-strand breaks and thereby to reduced cell death. However, reduced Top1 expression is probably only one among several resistance mechanisms to SN-38, as the Top1 level only changed marginally in the MCF-7_de novo_ cell line. Consistently, a recent study suggested elevated levels of Top1 as a predictive biomarker for irinotecan response in MBC patients [47]. The Top1 protein may be modified by several PTMs including phosphorylation [18, 30, 48], ubiquitination [49] and SUMOylation [50]. These modifications of Top1 have been associated to the response to Top1 targeting drugs and we therefore compared differences in the Top1 isoelectric patterns in the sensitive and resistant cell lines. We identified a novel pattern of the Top1 isoelectric patterns in the MDA_acq_ cells, which may contribute to the resistance to SN-38 (Fig. 3e).

## Conclusion

In conclusion, molecular characterization of the developed SN-38 resistant human BC cell line model system revealed that acquisition of resistance to SN-38 in vitro is multifactorial and that acquired or *de novo* resistance share fundamental characteristics. In particular, up-regulation of the BCRP drug efflux pump, low proliferation rates and down-regulation of Top1 protein are suggested as key mediators of SN-38 resistance in human breast cancer cell lines. These preclinical observations should be clinically validated in breast cancer biopsies derived from clinical studies in which the patients were exposed to Top1 inhibitor treatment to generate level 1 evidence for use of these markers.

## Additional files

Additional file 1:
**This file contains the following:** **Table S1**: a table with FISH data of *TOP1*/CEN-20 and *TOP2A*/CEN-17, *TOP1*/CEN-20 analyses of 52 human breast cancer cell lines. **Figure S1**: correlations between *TOP1* copynumbers from FISH and SNP analyses, and correlation between *TOP1* copy numbers and gene expression, presentation of 9 breast cancer cell lines with regards to *TOP1* copy numbers, ER and HER2 status and the sensitivity to SN-38. **Figure S2**: consistency between Top1 protein levels and *TOP1* copy numbers, Top1 enzymatic activity in 9 breast cancer cell lines and the influence of SN-38 on Top1 enzymatic activity, dose–response of 9 breast cancer cell lines to SN-38 and IC50 values. **Figure S3**: MTT data demonstration that the the acquired and de novo SN-38 resitant cell lines are resistant when grown in the concentration of SN-38 at which the the development of resistance was terminated. **Figure S4**: Growth curves of the SN-38 resistant cell lines in comparison to their parental and DMSO controls. **Figure S5**: Cell cycle distribution of resistant cell lines in comparison to their respective DMSO controls. **Figure S6**: Sensitivity to SN-38 in docetaxel resistant breast cancer cell lines. **Figure S7**: Global expression analysis showing sample clustering, the number of differentially expressed genes between resistant and wild type DMSO control cell lines and Enrichment of Gene Ontology Molecular Function. **Figure S8**: Western blotting of Top2a in SN-38 resistant cell lines in comparison to their respective controls. **Figure S9**: Nannocapillary analysis of Top1 and β-actin in SN-38 resistant MCF7 and MCF7 control cells. **Figure S10**: Western blotting of ID3 in SN-38 resistant MDA-MB-231 and MCF-7 cells and their controls cells. (PDF 757 kb) Additional file 2:
**32 genes that are differentially expressed in both the** **MDA-MB-231** **and the MCF7 model system.** (XLSX 4123 kb) Additional file 3:
**The top 50 overexpressed genes in the** **SN-38** **resistant** **MDA-MB-231** **cells.** (XLSX 61 kb) Additional file 4:
**The top 50 overexpressed genes in the** **SN-38** **resistant MCF7 cells.** (XLSX 62 kb)

## Abbreviations

BC: breast cancer
BCRP: breast cancer resistance protein
CEN17: centromere 17
CEN-20: centromere 20
CN: copy number
ER: estrogen receptor
FISH: Florescence in-situ hybridization
GO: Gene Ontology
HER2: human epidermal growth factor receptor 2
ID3: inhibitor of DNA binding 3
mBC: metastatic breast cancer
Pgp/MDR1: permeability-glycoprotein
PTM: Post translational modification.
SNP: single- nucleotide polymorphism
*TOP1*: topoisomerase I gene
Top1: topoisomerase I protein
