# Supplementary material for: Molecular characterization of irinotecan (SN-38) resistant human breast cancer cell lines
Source: BMC Cancer. 2016 Jan 22;16:34. doi: 10.1186/s12885-016-2071-1 (PMC4722663; doi:10.1186/s12885-016-2071-1)

Supplementary data

sFigure 1: FISH analysis of *TOP1* and CEN-20 in human breast cancer cell lines and the correlation between FISH/SNP derived *TOP1* copy numbers and between *TOP1* copy numbers and gene expression.
A) FISH analysis of *TOP1*, CEN-20 and *TOP1*/CEN-20 ratio in 52 breast cancer cell lines. Data are sorted from lowest to highest *TOP1* copy number. B) *TOP1* FISH score vs. SNP derived CN state of 52 breast cancer cell lines. Based on the *TOP1* SNP derived copy number values the cell lines were categorized as loss, neutral, gain or high gain. Kruskall-Wallis Test for trend across ordered groups $p<0.0001$. C) Correlation between *TOP1* FISH score and *TOP1* gene expression in 39 breast cancer cell lines (R^2=0.50, p=0.0012). D) Overview of the 9 selected cell lines with regards to ER, HER2, *TOP1* copy numbers and IC_{50} values

sFigure 2: Top1 protein expression, Top1 enzyme activity and sensitivities to SN-38 in 9 selected breast cancer cell lines.

A) Western blot showing the levels of Top1 in the 9 selected breast cancer cell lines. β-actin is included as loading control and the *TOP1* copy number status is indicated below the blot. B) Cellular Top1 enzyme activity in the 9 selected breast cancer cell lines and the response to 30 µM SN-38. Lysis buffer is included as a background control. C) The sensitivity to SN-38 and the derived IC_{50}values in the 9 selected breast cancer cell lines.

sFigure 3: Sensitivity of (a) Acquired and (b) *De novo* to their corresponding final concentration of SN-38 in comparison to their DMSO controls using MTT assay. Cell lines were exposed to their corresponding SN-38 concentration for 120h. Triplicate wells were analyzed, and data shown is mean ± s.d. of a representative experiment in percentage, n=3.

sFigure 4: Growth curves of the MDA_{acq}, MCF-7_{acq}, $MDA_{de\ novo}$, MCF-$7_{de\ novo}$ cell lines in comparison to their parental and DMSO controls. Cell lines were seeded in 6-well plates and each well was counted 3 times. Data shown is the mean of three counts of cells from each well ± s.d. Growth analysis was done without SN-38 added.

sFigure 5: Cell cycle distribution of resistant cell lines in comparison to their respective DMSO controls. Cells were stained with Propidium Idodide and analysed using FACS and FLOJO software. Cell cycle analysis was done in the absence of SN-38.

sFigure 6: Sensitivity to SN-38 in docetaxel resistant breast cancer cell lines. Docetaxel resistant MCF-7 (Res 65 nM) and MDA-231 (Res 150 nM) breast cancer cells lines and the corresponding parental control cell lines were exposed to SN-38 (0-30 µM) as indicated for 72 hours. Data shows relative viability as measured in MTT assays. Triplicate wells were analyzed, and data shown is mean ± s.d. of a representative experiment in percent of untreated cell lines.

sFigure 7: Global expression analysis (a) Sample clustering based on probe-level data show clear segregation of the two cell lines, but not MCF-7acq vs. MCF-7DMSO control. (b) Venn diagram illustrating the number of differentially expressed genes between resistant and wild type DMSO control cell lines. (c) Enrichment of Gene Ontology Molecular Function in differentially expressed genes in MCF-7acq cells (1) or MDAacq cells (2). The top 10 most enriched GO (molecular function) terms are shown. Bar length indicate the number of differentially expressed genes with the respective GO-term.

sFigure 8: Western blotting of Top2a in SN-38 resistant cell lines in comparison to their respective controls (Parental and DMSO). All samples were immunoblotted with an antibody to β-actin to illustrate equal protein loading (a) Acquired and (b) De novo

sFigure 9: Size analysis of Top1 and β-actin in acquired SN-38 resistant MCF7. Nanocapillary electrophoresis was applied to simultaneously detection and quantification of the Top1 and β-actin proteins.

sFigure 10: Western blotting of ID3 in MDA-MB-231 and MCF-7 cells with acquired resistence to SN-38 resistant cell lines in comparison to their respective controls (P: Parental, D: DMSO, R: Resistant). All samples were immunoblotted with an antibody to β-actin to illustrate equal protein loading.

Supplementary section

Tables

sTable 1. FISH analysis of *TOP1*/CEN-20 and *TOP2A*/CEN-17

Cell line	*TOP1* copy no.	CEN-20 copy no.	*TOP1*/CEN-20 ratio	*TOP2A* copy no.	CEN-17 copy no.	*TOP2A*/ CEN-17 ratio
**MDA_{acq}Parental**	2.6	2.18	1.22	2.48	2.15	1.16
**MDA_{acq}DMSO**	3.42	2.67	1.28	2.42	2.27	1.07
**MDA_{acq}**	3.2	2.73	1.17	2.83	2.45	1.16
**MCF-7_{acq} Parental**	3.75	2.87	1.31	1,83	2.05	0.89
**MCF-7_{acq} DMSO**	3.78	3.05	1.24	1.85	2.48	0.74
**MCF-7_{acq}**	3.52	2.73	1.29	1.75	2.08	0.84

sFigure 1

A

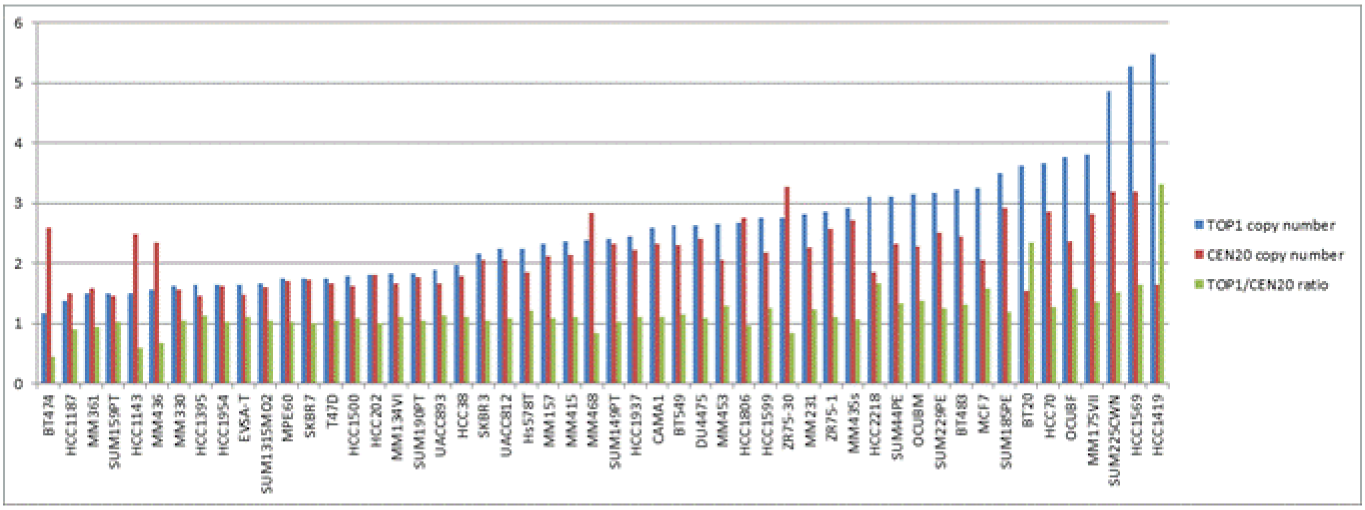

B

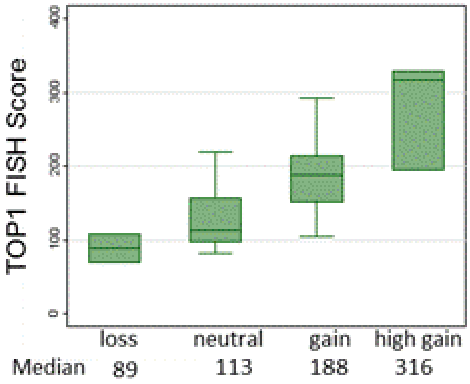

C

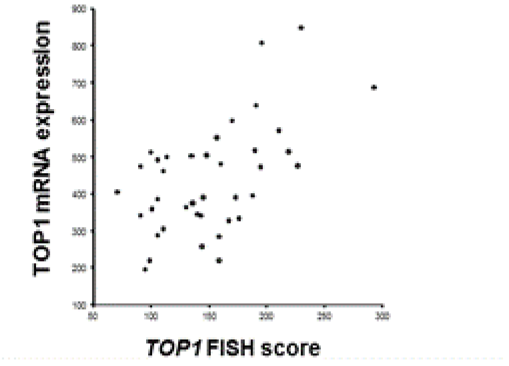

	HER2+		HER2 normal		
	ER+	ER-	ER+	ER-	
TOP1 CN Gain (IC50 values)	HCC1419 (181 nM)	HCC1569 (16 nM)	MCF-7 (74 nM)	HCC70 (64 nM)	MDA-MB-231 (248 nM)
TOP1 CN Loss or neutral (IC50 values)	BT474 (5127 nM)	HCC1954 (16 nM)	T47D (1 nM)	HCC1143 (93 nM)	

sFigure 2

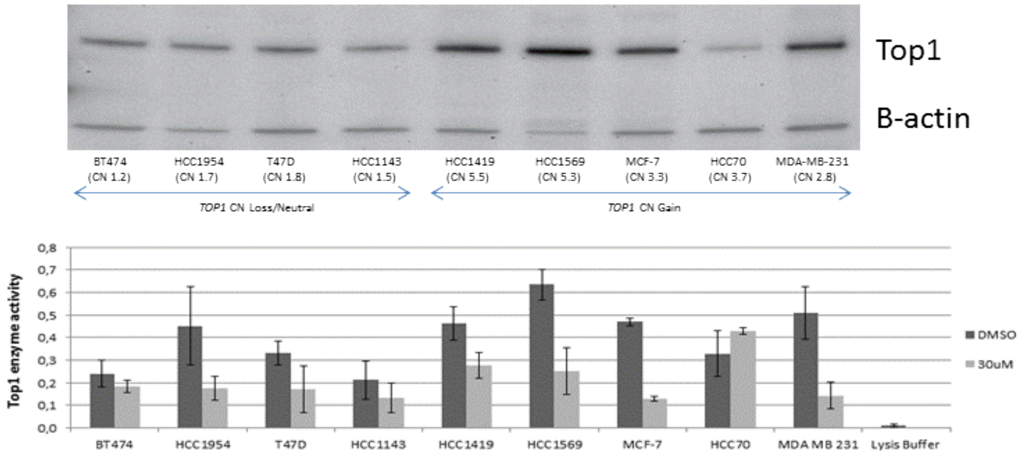

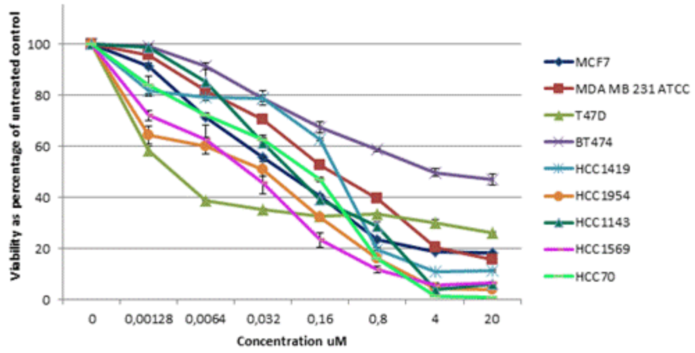

Cell line	IC50 (nM)
MCF7	73.7
T47D	1.1
BT474	5127.4
HCC1419	180.9
HCC1954	16.2
HCC1143	92.8
HCC1569	15.8
HCC70	64.2
MDA-MB-231	248.0

sFigure 3

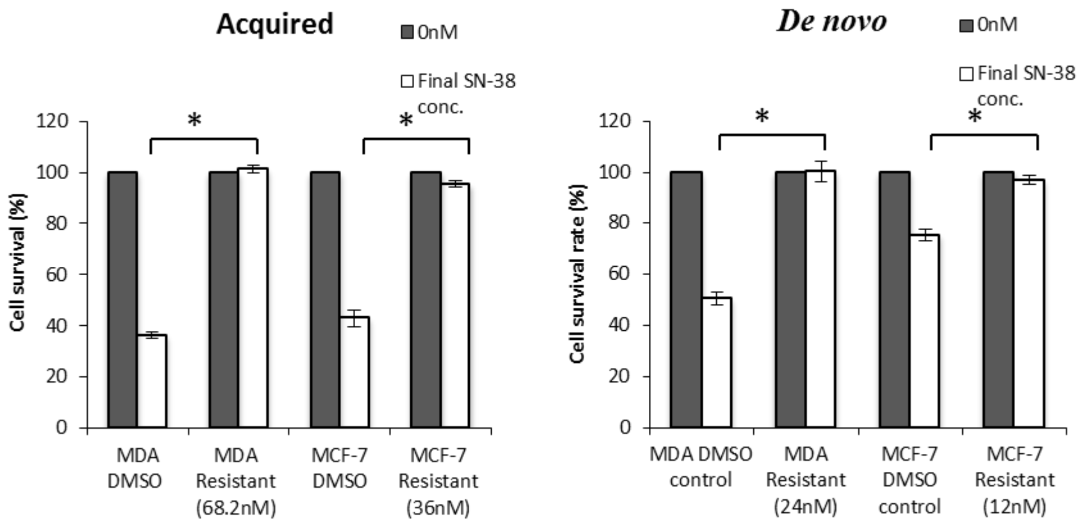

sFigure 4

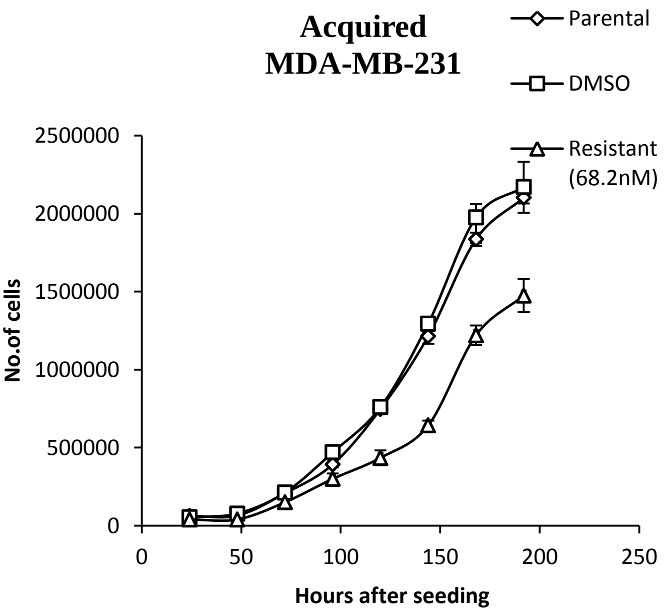

Acquired
MDA-MB-231
Parental
DMSO
Resistant
(68.2nM)
No.of cells
Hours after seeding

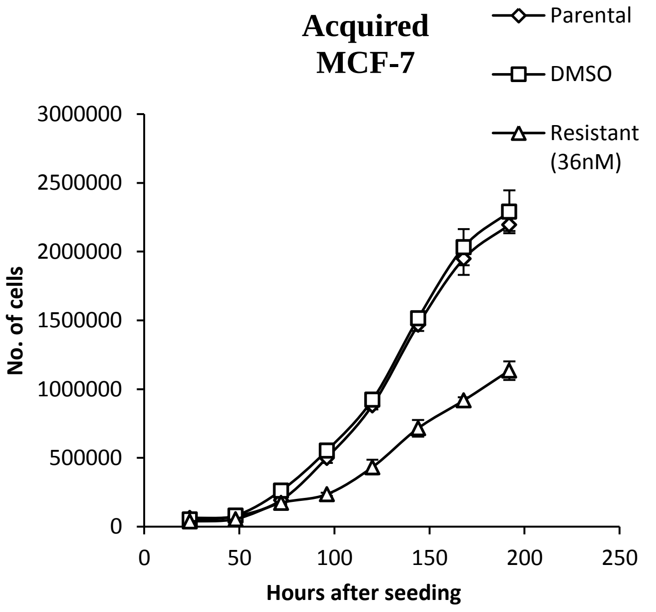

Acquired
MCF-7
Parental
DMSO
Resistant
(36nM)
No. of cells
Hours after seeding

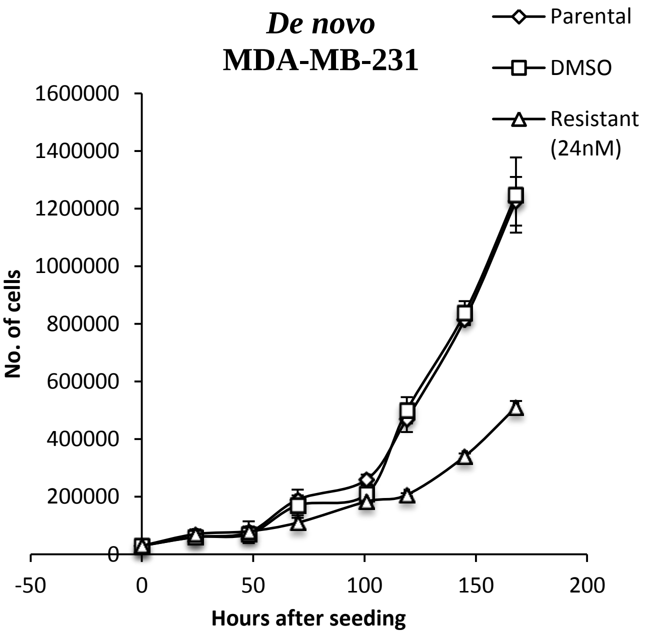

De novo
MDA-MB-231
Parental
DMSO
Resistant
(24nM)
No. of cells
Hours after seeding

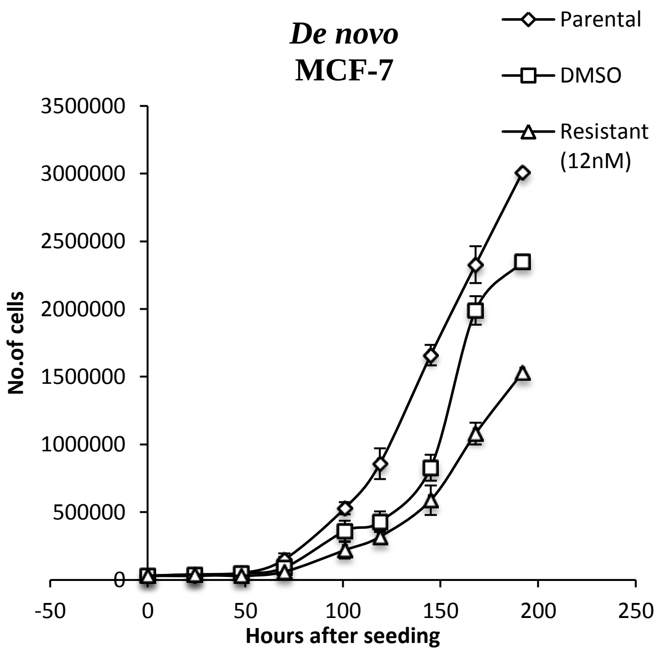

De novo
MCF-7
Parental
DMSO
Resistant
(12nM)
No.of cells
Hours after seeding

sFigure 5

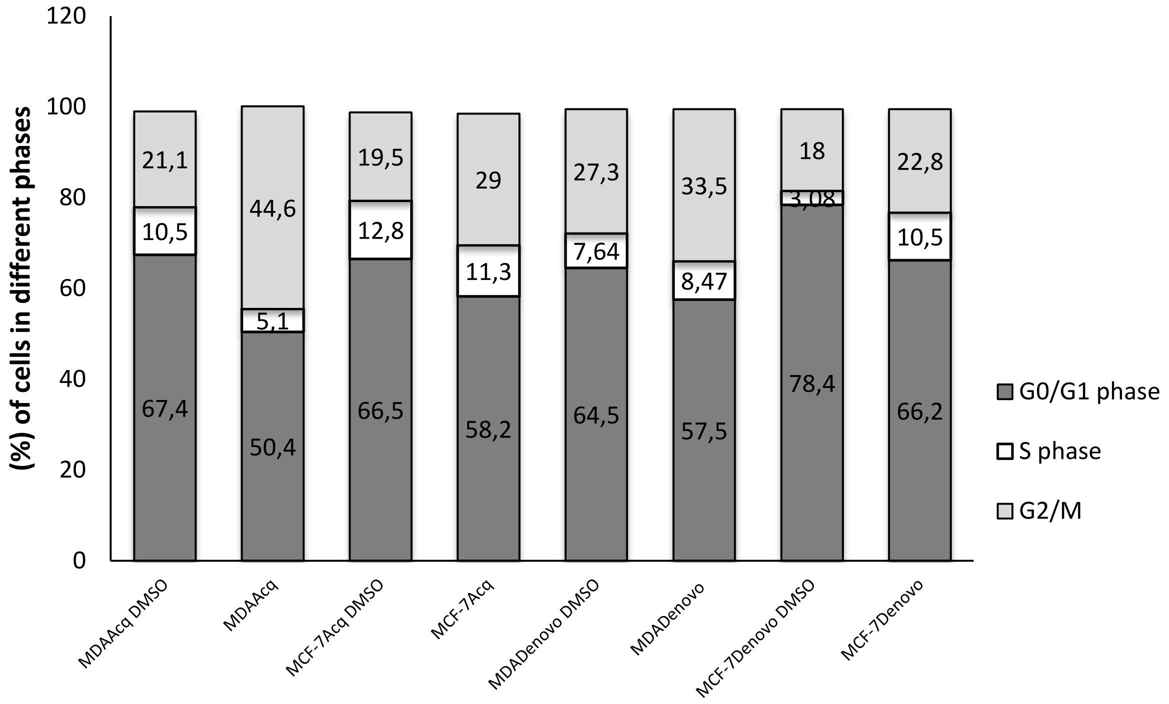
120
100
80
60
40
20
0
(%) of cells in different phases
21,1
10,5
67,4
44,6
5,1
50,4
19,5
12,8
66,5
29
11,3
58,2
27,3
7,64
64,5
33,5
8,47
57,5
18
3,08
78,4
22,8
10,5
66,2
MDAAcq DMSO
MDAAcq
MCF-7Acq DMSO
MCF-7Acq
MDADenovo DMSO
MDADenovo
MCF-7Denovo DMSO
MCF-7Denovo
G0/G1 phase
S phase
G2/M

sFigure 6

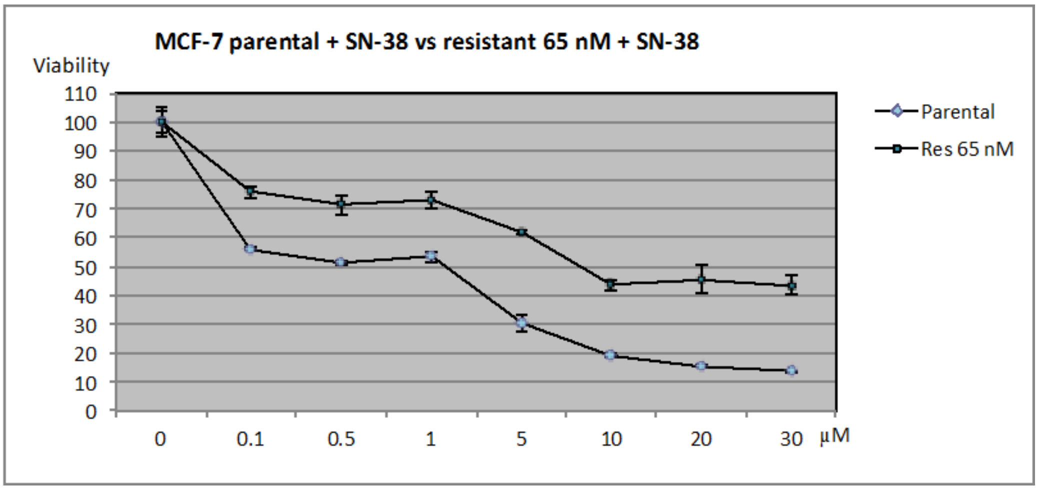
MCF-7 parental + SN-38 vs resistant 65 nM + SN-38
Viability
110
100
90
80
70
60
50
40
30
20
10
0
0
0.1
0.5
1
5
10
20
30
µM
Parental
Res 65 nM

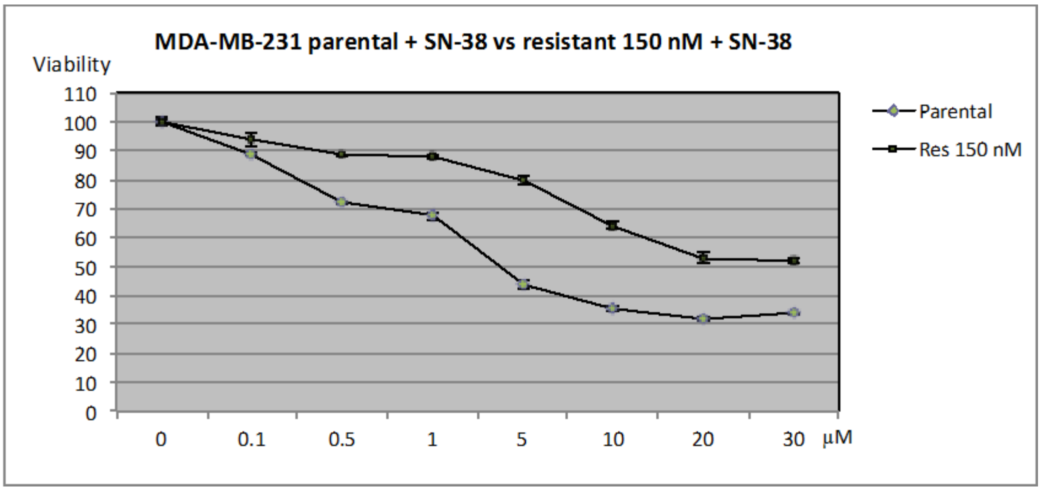
MDA-MB-231 parental + SN-38 vs resistant 150 nM + SN-38
Viability
110
100
90
80
70
60
50
40
30
20
10
0
0
0.1
0.5
1
5
10
20
30
µM
Parental
Res 150 nM

sFigure 7

(a)

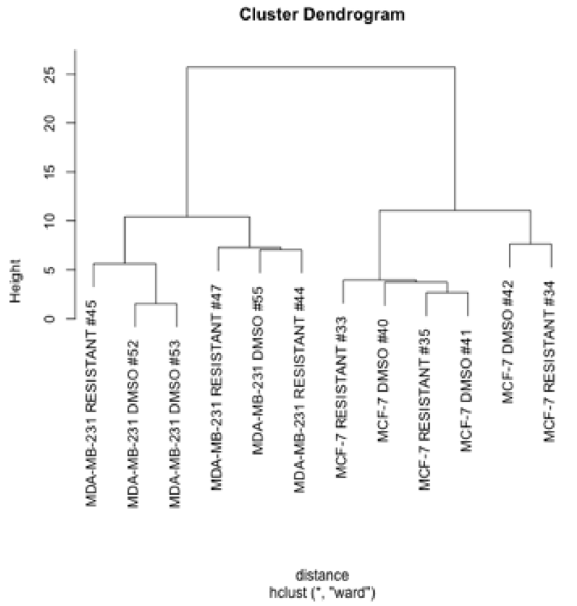

(b)

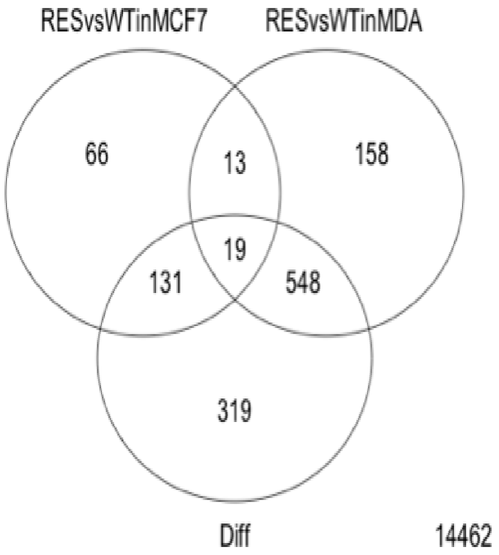

(c)

MCF-7$_{acq}$ vs. MCF-7$_{DMSO}$ control: Custom go

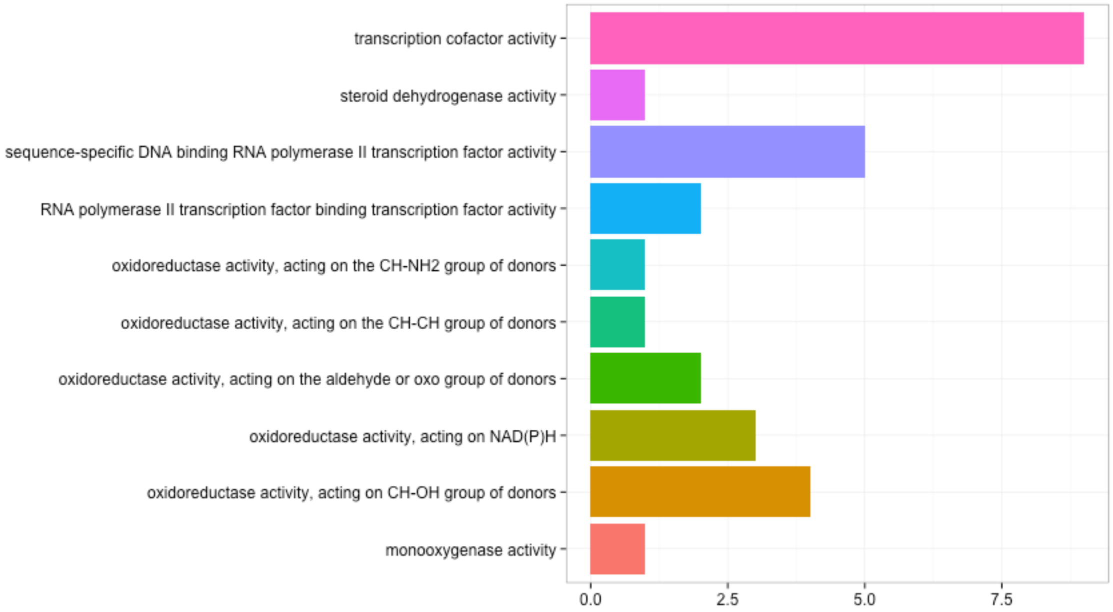

MDA_{acq} vs. MDA_{DMSO} control: Custom go

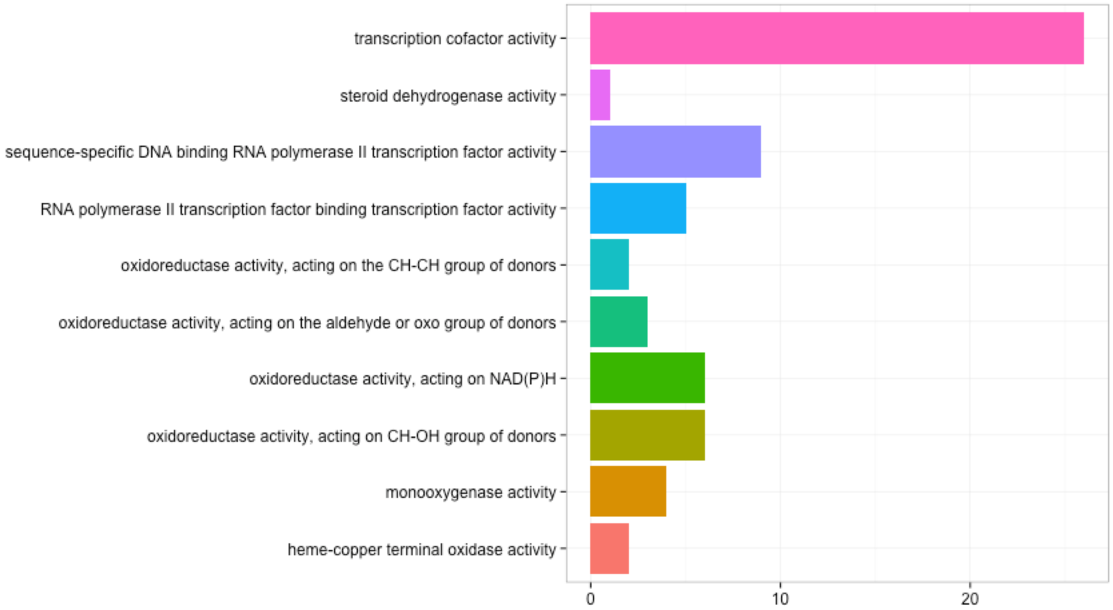

sFigure 8

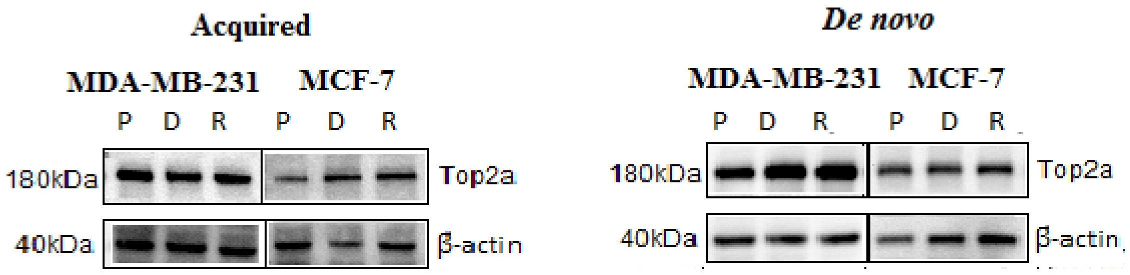

sFigure 9

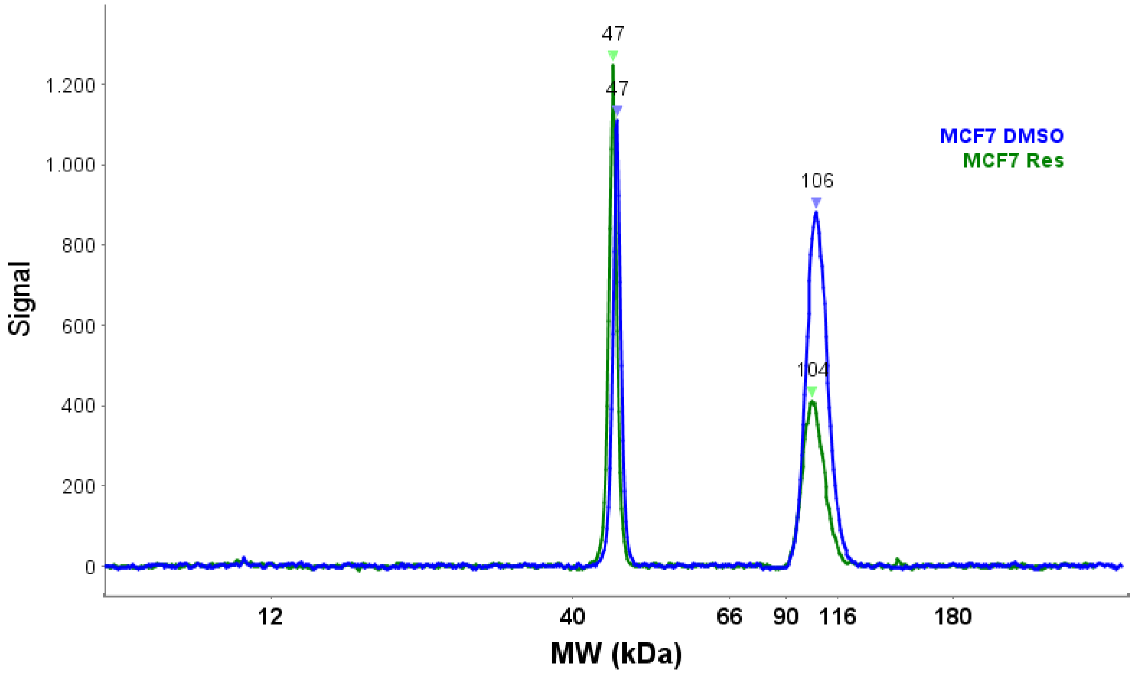

sFigure 10

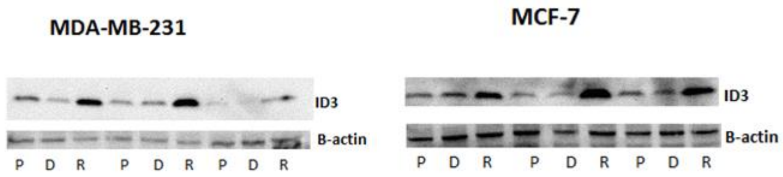

Supplement: Additional file 1: — This file contains the following: Table S1: a table with FISH data of TOP1/CEN-20 and TOP2A/CEN-17, TOP1/CEN-20 analyses of 52 human breast cancer cell lines. Figure S1: correlations between TOP1 copynumbers from FISH and SNP analyses, and correlation between TOP1 copy numbers and gene expression, presentation of 9 breast cancer cell lines with regards to TOP1 copy numbers, ER and HER2 status and the sensitivity to SN-38. Figure S2: consistency between Top1 protein levels and TOP1 copy numbers, Top1 enzymatic activity in 9 breast cancer cell lines and the influence of SN-38 on Top1 enzymatic activity, dose–response of 9 breast cancer cell lines to SN-38 and IC50 values. Figure S3: MTT data demonstration that the the acquired and de novo SN-38 resitant cell lines are resistant when grown in the concentration of SN-38 at which the the development of resistance was terminated. Figure S4: Growth curves of the SN-38 resistant cell lines in comparison to their parental and DMSO controls. Figure S5: Cell cycle distribution of resistant cell lines in comparison to their respective DMSO controls. Figure S6: Sensitivity to SN-38 in docetaxel resistant breast cancer cell lines. Figure S7: Global expression analysis showing sample clustering, the number of differentially expressed genes between resistant and wild type DMSO control cell lines and Enrichment of Gene Ontology Molecular Function. Figure S8: Western blotting of Top2a in SN-38 resistant cell lines in comparison to their respective controls. Figure S9: Nannocapillary analysis of Top1 and β-actin in SN-38 resistant MCF7 and MCF7 control cells. Figure S10: Western blotting of ID3 in SN-38 resistant MDA-MB-231 and MCF-7 cells and their controls cells. (PDF 757 kb) [file 12885_2016_2071_MOESM1_ESM.pdf]
